# A smart look at monitoring while drilling (MWD) and optimizing using acoustic emission technique (AET)

**DOI:** 10.1038/s41598-024-70717-8

**Published:** 2024-08-26

**Authors:** Mehrbod Khoshouei, Raheb Bagherpour, Mojtaba Yari

**Affiliations:** 1 Department of Mining Engineering, Isfahan University of Technology, Isfahan, 8415683111 Iran; 2 Department of Mining Engineering, Faculty of Engineering, Malayer University, Malayer, Iran

**Keywords:** Rock drilling, Specific energy (SE), Acoustic emission technique (AET), Acoustic and vibration signals, Real-time monitoring, Environmental sciences, Energy science and technology, Engineering

## Abstract

Monitoring while drilling (MWD) is a crucial task in mining operations. Accurately measuring drill and rock-related operating parameters can significantly reduce the cost of drilling operations. This study explores the potential of monitoring drilling specific energy (SE) and optimizing drilling operations by processing vibroacoustic signals generated while drilling. For this purpose, 30 samples of different rocks, are used for drilling tests. During the drilling process, the acoustic and vibration signals are recorded and analyzed in the time, frequency, and time–frequency domains., and parameters related to the resulting spectra are extracted. After obtaining the vibroacoustic parameters for drilling, the relationship between them and the drilling SE was investigated. There is evidence that the progression of SE contributes to the magnitude of rock drilling vibroacoustic features, which could be employed to indicate energy conditions during drilling. Results obtained in this study have the potential to be used as the basis for an industrial monitoring system that can detect excessive energy consumption and advise the user of the end of the bit's useful life. This method can be an intelligent technique for measuring the behavior of real-time drilling operations based on the SE simply by installing vibroacoustic sensors on the drilling machines.

## Introduction

Drilling operation is one of the most critical and expensive parts of exploration, extraction, and production of minerals, oil, and gas. More than 25–35% of mining operation costs are related to drilling. Various factors influence drilling costs, including deep excavations, complex drilling designs, extensive exploration operations, and complex geological conditions. Therefore, assessing drilling performance should be included in the feasibility and economic analysis of any project^1–4^. Drilling is a complex process with various factors that affect its performance^5–7^. The parameters affecting the drilling operation include three general categories of parameters related to the drilling machine and cutting tool, rock properties, and operational or technical parameters^8,9^. The combination of various parameters can significantly impact the performance of drilling operations. However, predicting and evaluating the outcomes of such complex conditions often becomes challenging. Therefore, it is essential to have a comprehensive method or an indicator that can accurately measure the impact of these factors or monitor the operation without disrupting the ongoing operation. Such a method can be highly beneficial in monitoring and optimizing the drilling process.

Basically, monitoring of drilling condition avoids unexpected changes, reduces energy consumption, and eventually reduces production costs and labor^10,11^. Cost and energy optimization and productivity became the main purpose in operation monitoring^12^. Based on field experience, there are many ways to optimize drilling operations and reduce costs^13^. One of these methods is the optimization of drilling parameters to achieve minimum Specific Energy (SE).

The SE is a useful tool to determine whether a system is operating efficiently or not. When a drill bit is functioning at its highest efficiency, the energy-to-rock volume ratio stays relatively constant^14^. This relationship is used in drilling operations to determine whether SE changes while adjusting different drilling parameters such as WOB (Weight on Bit) or rotational speed. If the SE remains constant while increasing the WOB, the bit is still efficient. However, if the SE increases significantly, the bit needs to be replaced^15,16^. This helps drillers to determine the most appropriate parameters when redesigning the drilling system. To optimize drilling through energy, the first step is to accurately predict and evaluate the drilling energy in real time. Since evaluating and predicting the drilling SE is vital, several studies have been conducted to predict and calculate the SE using various drilling parameters such as operation parameters and rock properties.

This study significantly contributes to drilling monitoring by comprehensively analyzing vibroacoustic signals in the time, frequency, and time–frequency domains to understand SE. The research emphasizes the potential for real-time monitoring and optimization of drilling operations through continuous vibroacoustic signal tracking, establishing a clear relationship between SE and vibroacoustic features. This novel approach provides a robust framework for using acoustic emission (AE) as a predictive tool for drilling efficiency, advancing the understanding and capabilities of condition monitoring systems.

### Specific energy (SE) of drilling

**Fig. 1:**
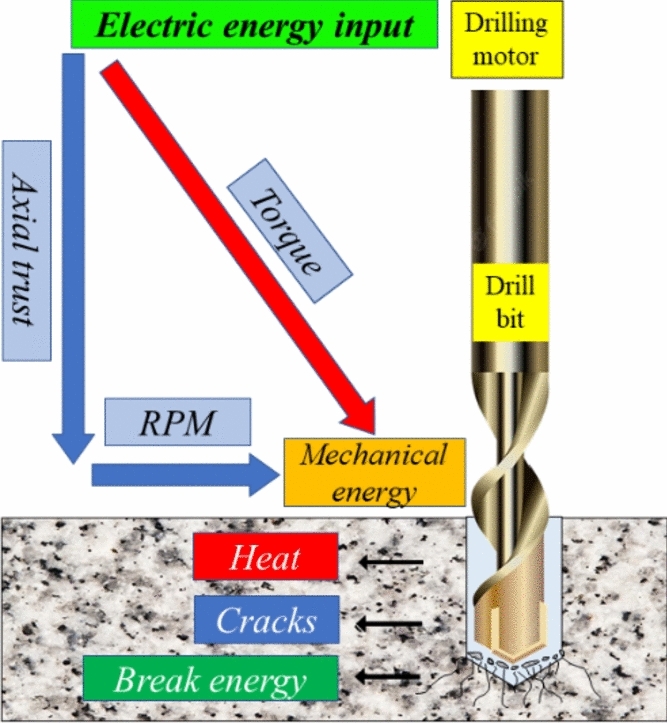
The converting input energy into rock fracture energy in rock drilling operations^19^.

In rock drilling, there are many stresses in the interaction between the drill bit and the rock due to the rotation and compressive force applied to the drill bit. The rock absorbs the energy produced by the drilling machine. In this process, some of the energy passes through the rock, creating cracks and fractures, ultimately leading to a fractured zone, allowing the rock to be drilled. Additionally, part of the energy is also reflected^17,18^. Through evaluating the drilling operation mechanism, as shown in Fig. 1, it is evident that the drilling energy is dissipated at different stages of drilling^19^. The energy applied to the drill bit causes the following effects:

1. Breaking the rock, which is a desirable outcome^20^.
2. Development of radial cracks and heat, which are caused by a part of the input energy.

Various experimental relationships have been developed to calculate and predict drilling energy^21,22^. These relationships use different operational parameters, such as WOB, drill bit speed, torque, etc., to calculate drilling SE. In this section, some of the critical and widely used relationships are presented. Teale introduced the concept of drilling SE in 1965 to quantify the energy needed to destroy a particular volume of rock. According to Teale's definition, SE is calculated as the energy required to remove a given volume of rock, as described in Eq. (1)^14–25^.

1 $$SE=\frac{Total required energy}{Removed rock volume}$$

Calculating the volume of a drill hole involves multiplying the cross-sectional area by the depth of penetration (Δh). In addition, energy can be defined as the amount of force applied multiplied by the distance. During the drilling process, two forces are applied to the drill bit: weight on the bit (axial force) and torque (rotational force). Taking all these factors into account, Eqs. (2) and (3) are derived.

2 $$SE=\frac{ Axial force }{ Volume of rock}+\frac{Rotational\text{ force}}{Volume of rock}$$

3 $$SE=\frac{WOB\times \Delta h}{Area\times \Delta h}+\frac{Torque\times 2\pi \times \# of rotations}{Area\times \Delta h}$$

WOB is the drill bit's weight (lbf), and ∆h is the penetration depth (ft). Calculating SE requires considering various factors, including the type of drilling operation (e.g., rotary drilling, percussion drilling), the rock type being drilled, the weight on the bit, the rotational speed, and the penetration rate. Several equations and models have been developed to calculate specific energy, which shows the importance of calculating the SE in the drilling process.

Continuous energy efficiency monitoring has proven to be a valuable tool in determining the cause of ineffectiveness. It is widely used in the drilling industry, as stated by Dupriest and Koederitz in 2005^15^. This concept has been used as a powerful ROP management tool in many previous field management studies. Some projects have used this concept on real-time drilling data, while others have used it as a basis for ROP management before starting the drilling operation. In recent years, this idea has proven to be an effective method for managing ROP in various field management studies. Some projects have utilized this approach on drilling data in real-time, while others have used it as a foundation for ROP management before commencing the drilling process.

In 1991, Göktan developed a correlation between the SE observed during microscale laboratory cutting tests. However, it was concluded that no reasonable relationship can be established between the brittleness index and SE^26^. In 1992, Pessier and Fear developed a new mathematical equation for the penetration rate based on the SE equation derived by Teale^27^. In 1996, Reddish and Yasar investigated the rock toughness index test based on the SE of the drilling process. It was concluded that the rock strength index was more reliable than other index tests, and the test results can be applied practically in the field^28^.

Copur et al. searched for relationships between optimum SE obtained by full-scale cutting tests and compressive and tensile strength^24^. They also explored the correlation between a brittleness index obtained from macro-scale indentation tests and the efficiency of rock cutting, which includes SE and cutter forces^29^. In another study, Atici and Ersoy statistically analyzed the relationship between the brittleness and cutting SE of diamond saw blades and the drilling SE of compact polycrystalline diamond (PDC) bits^30^ .

Curry et al. developed an algorithm to estimate the technical limits of mechanical SE from the sonic log, lithology, and pressure data^31^. Dupriest et al. used the concept of SE to make operational decisions by using real-time data from the drilling site and designing an intelligent system for this purpose after the drilling operation.^32^. Remmert et al. followed the study of Dupriest et al. and detected the limiting factors of SE in ROP management projects^33^. Armenta used the Drilling SE concept as a modified version of the mechanical SE concept for ROP management in two test wells. The concept was used for the prediction of several drilling parameters such as ROP and also for detecting some particular situations like bit balling that require the proper reaction from the drilling crew^34^. Amadi and Iyalla performed an ROP management study in a deep-water oil field by using the SE concept. In this project, the optimum values of ROP are predicted from the real-time drilling data and intrinsic formation information^35^. Alali et al. developed an axial oscillation tool using the MSE concept to improve the transition of WOB to the bit, decrease the bit torque and drag, and increase ROP. This tool produces several pressure pulses that act on the pump open area of a shock tool to generate axial motions required to reduce frictions and downhole torques, resulting in more drilling performance by reducing the pipe buckling and bit torque and increasing ROP and bit life^36^.

Mohan et al. utilized the hydraulic mechanical specific energy (HMSE) concept, a modified version of SE, to manage ROP. This approach resulted in a more realistic representation of drilling efficiency in the tested wells^37^. Pinto and Lima carried out a study to optimize drilling operations in Brazilian saltwaters using the concept of SE. They utilized minimum and maximum limiting bounds to control ROP based on the values of SE and uniaxial compressive strength (UCS). This resulted in increased drilling efficiency and reduced failure of the bottomhole assembly (BHA)^38^. Al-Kindi et al. in 2017 utilized SE to aid the decision-making process while drilling, primarily to identify optimal pull-out-of-hole timing and prevent ROP decline^39,40^. Suppes et al. have developed a new application for the SE concept. They have increased the average and maximum ROP by monitoring SE in casing milling operations. The values of ROP in casing milling operations are correlated to the amounts of SE by a newly developed^41^. In 2021, Kolapo investigated the impact of mechanical rock properties on SE and ROP. Several empirical correlations were also developed based on laboratory tests to calculate ROP in various formations using UCS and tensile stress^42^. The novelty of the method of this study lies in its comprehensive signal analysis across time, frequency, and time–frequency domains. This approach gives a complete understanding of acoustic and vibration characteristics during drilling. Unlike traditional methods that rely on post-process analysis, this approach includes real-time monitoring and optimization capabilities. Additionally, the study establishes a direct link between SE and vibroacoustic features, presenting AE as a predictive tool for drilling efficiency, an area less explored in previous studies.

### Acoustic emission technique (AET)

Numerous attempts have been made to find a fast, accurate, non-destructive, and highly reliable method to monitor the condition of rock drilling operations. One such method is the use of acoustic waves. Acoustic waves are pressure waves generated by the release of energy during deformation or fracture, which pass through materials at the speed of sound. These waves can be detected by sound sensors^5,43,44^. Acoustic waves possess several characteristics like amplitude, wavelength, frequency, phase, wave energy, intensity, and pressure. These characteristics can be utilized to identify materials, estimate their properties, or monitor operational conditions. In recent times, acoustic waves have gained significant popularity in predicting material properties, monitoring, and detection applications.

One of the most crucial applications of acoustic waves is predicting the energy consumption, and wear rate of drill bits during drilling operations^45,46^ or ROP^47^. AE signals, along with other sensors like temperature and current sensors, are integrated into turning processes to monitor the energy consumption and enhance tool life^12^.

Between 2018 and 2019, Yari et al. conducted a series of laboratory drilling tests to estimate rock properties such as porosity, P-wave velocity, UCS, and Brazilian Tensile Strength (BTS) using acoustic emission testing. Their research found that rock properties can be predicted by analyzing the dominant frequencies (frequencies with the highest energy intensity)^9,8,48^. In 2020, Khoshouei and Bagherpour predicted three mechanical properties of hard rocks during laboratory-scale drilling by investigating the relationship between UCS, BTS, and Schmidt Rebound Number (SRN) with acoustic parameters such as Sound Pressure Level (SPL), First Dominant Frequency (FDF), and Vibration Level (VL). Statistical analysis of the results showed that there was a significant correlation between the rock properties and the properties of acoustic and vibration signals during the rock drilling process^49,50^.

Flegner et al. conducted a study on the vibroacoustic signals that are produced during rotary drilling. In their research, they analyzed the vibroacoustic signals in both the time and frequency domains. The histograms of the vibroacoustic signals in the time domain were used to classify different rock types. On the other hand, the vibroacoustic signal spectra in the frequency domain were used to determine the dynamic properties of the drilling operation^51^.

Acoustic waves have a wide range of uses, including detection^52^, Wang et al. demonstrated that acoustic sensors can detect properties of solid particles like sand and gravel in liquid–solid transfer systems. The study showed that particle size and flow rate impacted signals^53^. In addition, acoustic waves can be used to identify rock types during drilling operations. Qin et al. identified rock types during drilling operations by processing the signals in the frequency domain using the Short-Time Fourier Transform (STFT)^54^. Many other studies have also been performed on applying vibroacoustic wave processing for predicting the properties of materials or monitoring and detecting them.

The advantages of using acoustic signals are numerous. AE is highly sensitive, enabling the early detection of subtle changes and anomalies. The ability of AE systems to capture and process data in real time provides immediate insights for dynamic adjustments and decision-making. Additionally, AE's versatility across different materials and environments makes it valuable for various industrial applications. The study demonstrates AE's high accuracy and reliability in predicting SE, which is crucial for precision in industrial settings. By leveraging these unique capabilities, this study sets the stage for more efficient and effective real-time monitoring systems in drilling operations.

The present study investigates the use of vibroacoustic waves in monitoring the drilling SE. For this purpose, after collecting groups of rock samples (including granite, marble, and travertine) and preparing them for drilling tests, they are drilled by a laboratory drilling machine, and the vibroacoustic signals generated during drilling are recorded. After pre-processing, the signals are analyzed in time and frequency domains using the Fast Fourier Transform (FFT), STFT, and wavelet analysis methods. By extracting the features of the signals, the relationships between them and the SE of rock drilling tests were investigated.

## Experimental work

### Laboratory scale drilling machine

This study involved drilling tests conducted using a small-scale rotary drilling machine. Each rock sample was drilled under identical operating conditions to eliminate external factors that could influence the recorded vibration and acoustic signals. This ensured that the properties of these signals were solely dependent on the drilling operating parameters. The details of the operating conditions are summarized in Table 1.

**Table 1:** Operating conditions of drilling tests.

Title	Description
Drilling machine	Laboratory scale rotary drilling machine
Drill bit	V-type drill bit
Rotational speed	830 RPM
Drill bit diameter	8 mm
Drill bit type	Diamond bit Special for hard rocks
Drilling fluid	Cool water

### Rock samples

**Fig. 2:**
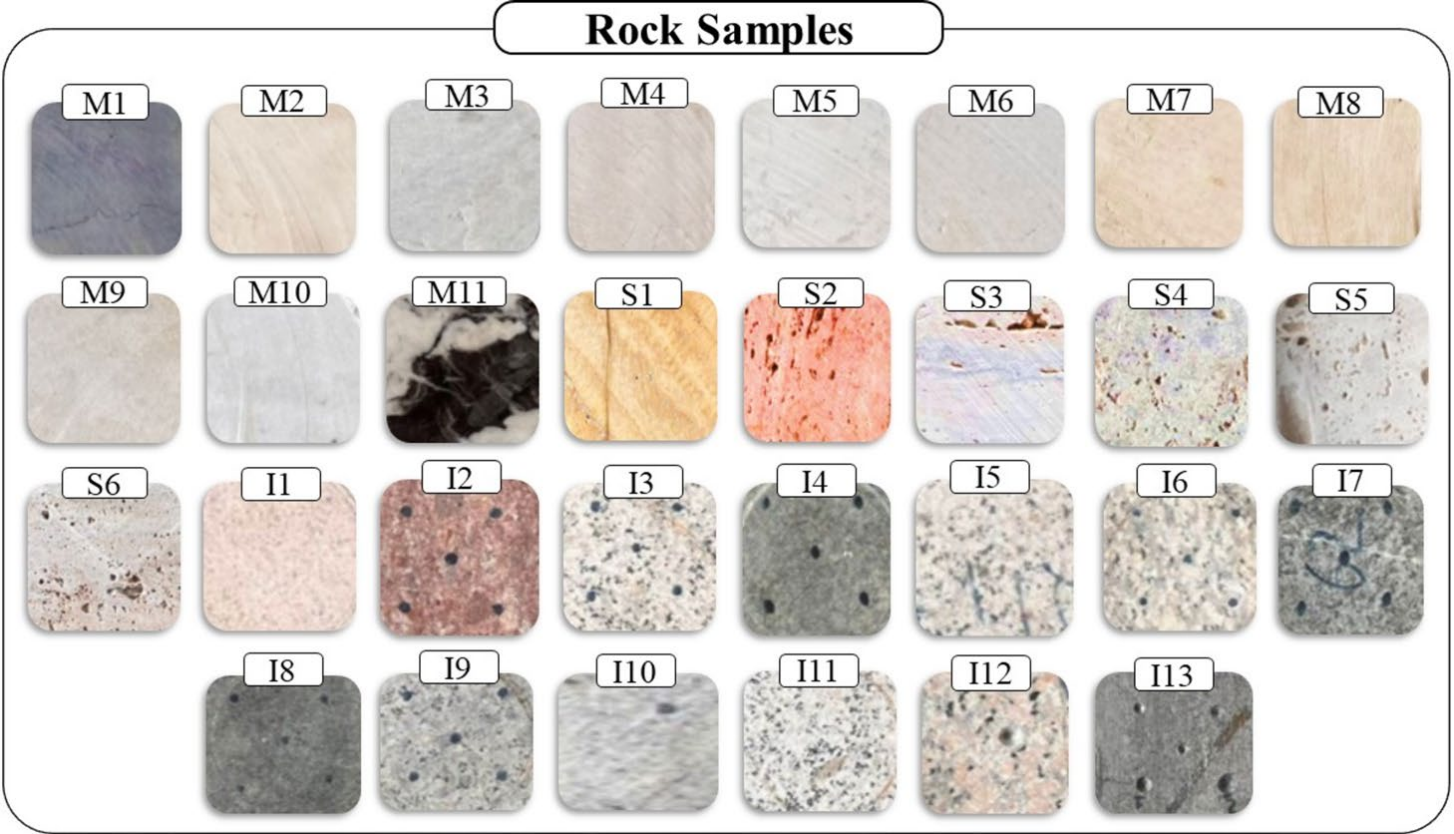
The tasted rock samples.

To study the effect of Energy consumption behavior and penetration rate on the characteristics of vibroacoustic signals generated during drilling, 30 rock samples with different lithological origins, including granite, marble, and travertine rocks in a wide range of physical, mechanical, and microscopic characteristics, were selected and prepared for the tests. These samples were selected based on their varying physical and mechanical properties to represent a wide range of typical drilling conditions. Figure 2 shows the tasted rock samples. The rock samples were collected from different mines and prepared for drilling tests. Table 2 shows the identification code of the rocks, rock type, and commercial names of rock samples.

**Table 2:** Identifier code, the type, and the commercial name of rock samples.

No	Code	Rock type	Commercial name	No	Code	Rock type	Commercial name
1	I1	Granite	Takab	16	M3	Marble	Laybid
2	I2	Red Granite	Yazd	17	M4	Marble	Salsali
3	I3	Granite	Natanz	18	M5	Marble	Partavoosi
4	I4	Diorite	Yashmi Birjand	19	M6	Marble	Harsin
5	I5	Granite	Sefid Natanz	20	M7	Marble	Tabas
6	I6	Granite	Nehbandan	21	M8	Marble	Kermanshah
7	I7	Diorite	Piranshahr	22	M9	Marble	Semirom
8	I8	Diorite	Meshki Molaee	23	M10	Marble	Chini Shiraz
9	I9	Granite	Borujerd	24	M11	Marble	Anarak
10	I10	Granite	Sefid Esfahan	25	S1	Travertine	Esfahan
11	I11	Granite	Saqez	26	S2	Travertine	Limoui Esfahan
12	I12	Granite	Neyriz	27	S3	Travertine	Takab
13	I13	Diorite	Meshki Natanz	28	S4	Travertine	Asali
14	M1	Marble	Lashotor	29	S5	Travertine	Dare- Bokhari
15	M2	Marble	Islam Abad	30	S6	Travertine	Abbas abad

After collecting the rock samples, they were cut into cube-shaped blocks with 10 cm dimensions and were prepared for drilling tests.

### Data recording equipment in drilling tests

During the drilling tests, three types of signals were recorded. The first signal was the acoustic signal produced from the point of impact of the drill bit with the rock. This signal was captured by a condenser microphone. The second signal was the ambient sound pressure level (SPL) generated in the drilling laboratory environment, which was recorded by a digital sound level meter. Lastly, the vibration signal generated from the vibration on the axis of the drilling machine during the operation was captured by a 3-axis accelerometer. A summary of the specifications of the signal recording equipment used in this study is provided in Table 3.

**Table 3:** Specifications of the data collection (signal recording) equipment.

Title	Description	Sampling frequency	Figs
Acoustic sensor	Acoustic condenser microphone, model: AR 321	44,100 Hz	
Sound Pressure Level meter	SPL meter, model: GM1356	1 Hz	
Vibration sensor	3-axis acceleration sensor	50 Hz	

**Fig. 3:**
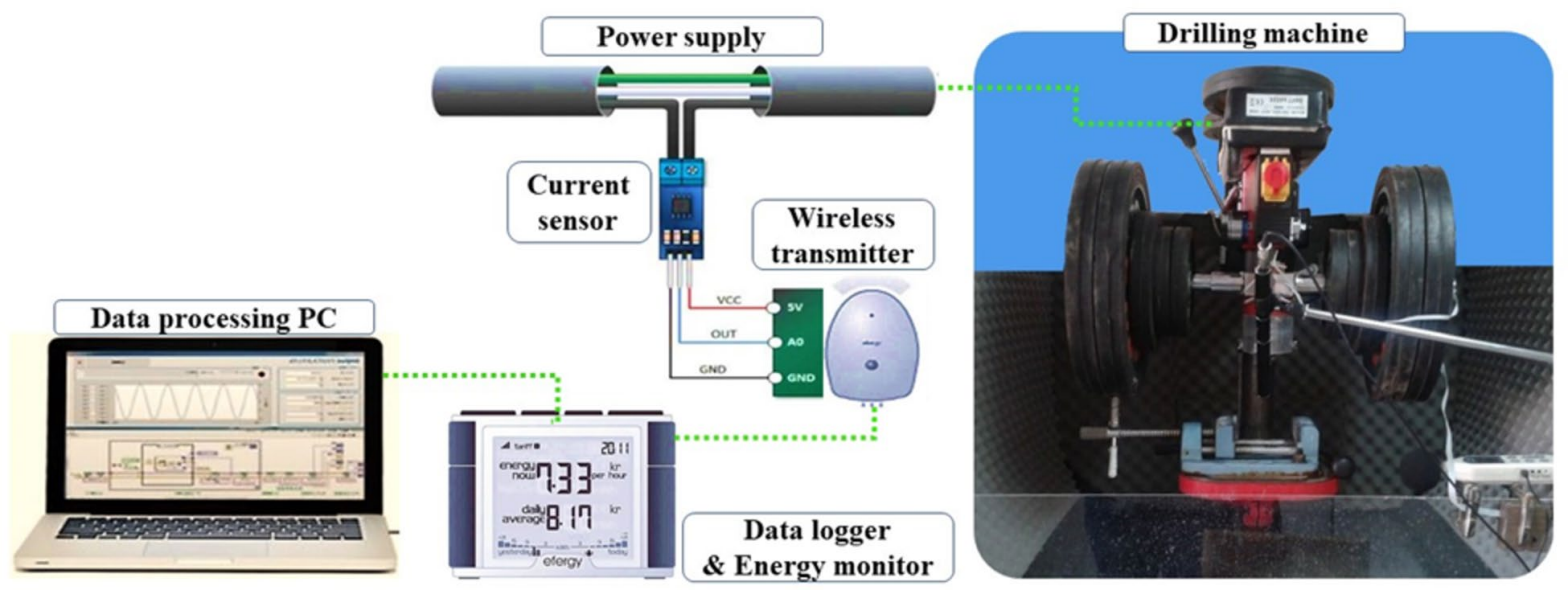
Experimental process of SE measuring and related equipment^19^.

The SE of rock drilling tests was obtained directly by installing the sensor of the electrical energy consumption (current sensor) of the drilling machine and dividing the amount of electrical energy consumed during drilling by the volume of the drilled rock during each test according to Eq. (1). The process and equipment for collecting and recording drilling energy are shown in Fig. 3.

**Fig. 4:**
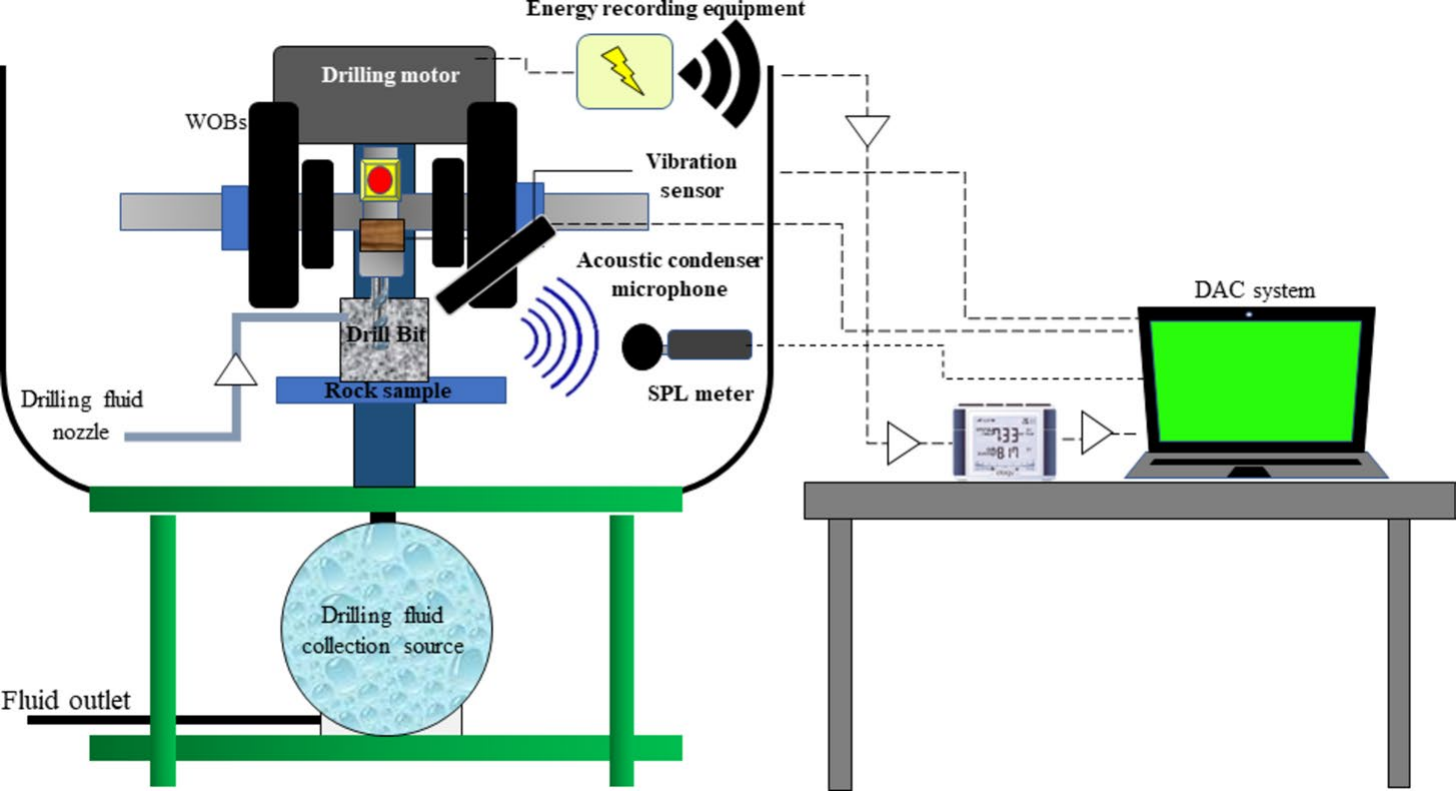
A view of the position of signals and energy recording equipment (obtained from^19^).

The position of the signal recording equipment on the drilling machine and the general signal processing procedure are shown in Fig. 4. The microphone is placed near the place where the drill bit collides with the rock surface. The sound level meter is set at a constant distance from the drilling machine in the acoustic chamber, and the vibration sensor is fixed on the machine's axis and aligned with the drill bit.

**Fig. 5:**
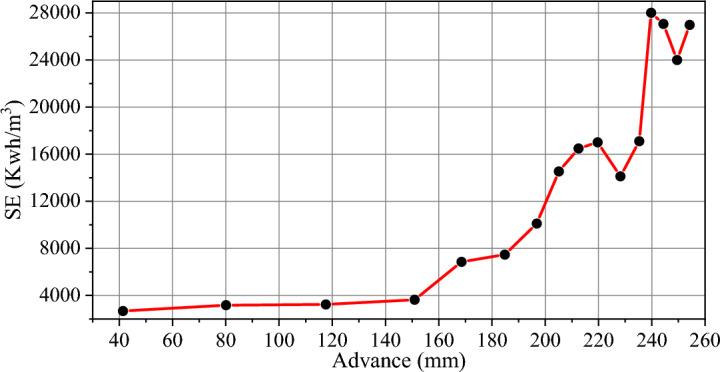
The SE during the granite rock sample # 1 drilling.

It has been observed that the SE of rock drilling exhibited a sudden increase during the life of the drill bit, after conducting drilling tests and calculating the values of SE. This conclusion was drawn by analyzing the graphs which illustrate the energy behavior. An example of such a trend is demonstrated in Fig. 5 which displays the SE trend during the drilling of a granite sample with high abrasivity (Sample #1).

**Fig. 6:**
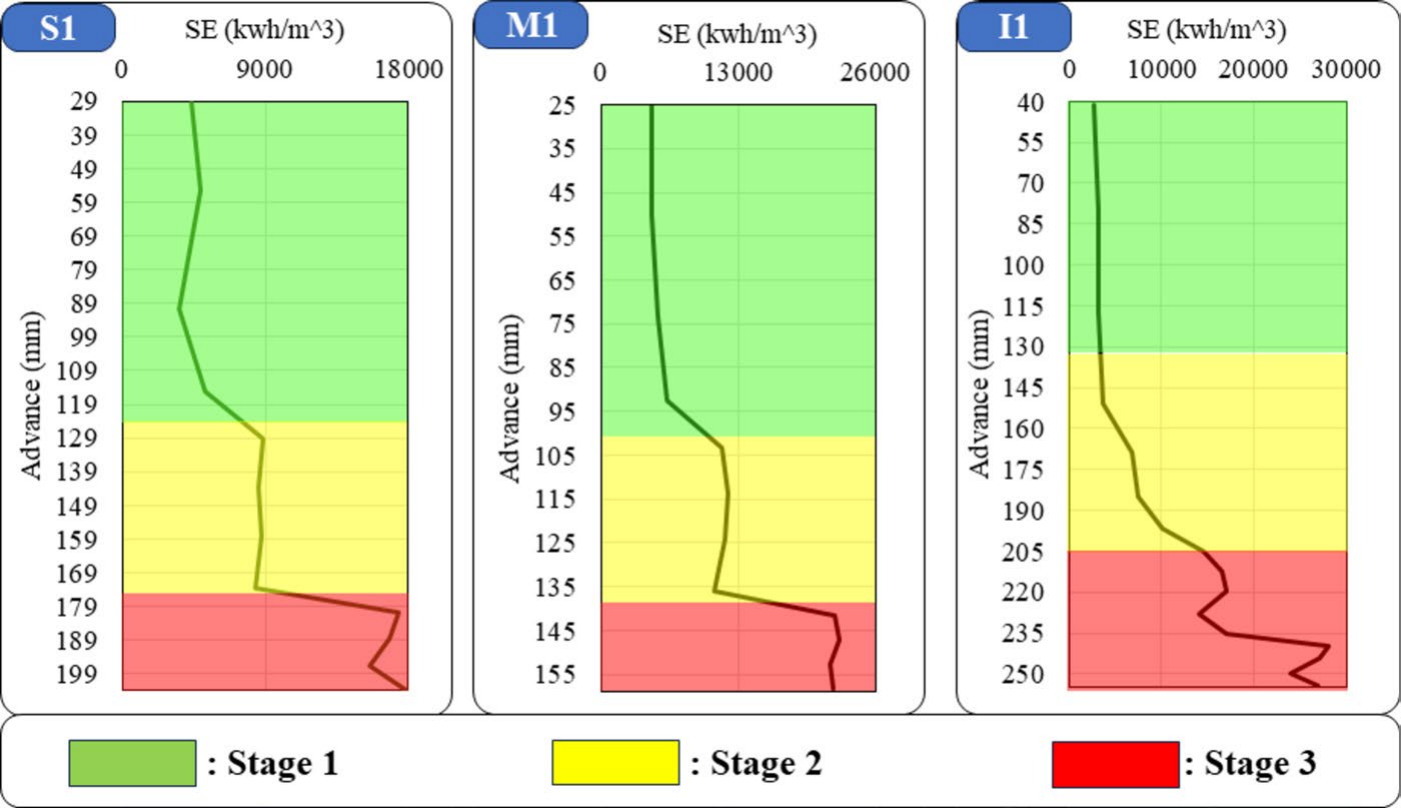
Changes in SE during the drilling of rock samples in three groups of granite, marble, and travertine rocks.

By examining the trend of drilling SE, three phases can be identified during the lifetime of the drill bit. The first stage is the new drill bit (optimized drilling); in this stage, due to the newness of the bit, the energy consumption is low. With the progress of drilling and further penetration into the rock, SE gradually increases. With the progress of drilling and passing through the optimized state, energy consumption increases. At this drilling stage, the drill bit is also in a more stable condition. As it progresses further into the rock, the drill bit enters a stage where energy consumption suddenly increases rapidly (sudden increase in the slope of the energy graph). At this stage, the drill bit must be replaced because, on the one hand, the energy consumption is high; on the other hand, the drill bit is possible to break, get stuck in the rock, and stop the drilling. Such a trend was observed in the drilling of all rock samples. Figure 6 shows the change of SE in different groups of rocks during the advance of the drill bit.

This study conducted a total of 336 drilling tests on different types of rocks in a controlled laboratory environment. The tests were categorized based on the energy state of the drilling, which was optimized drilling (99 tests), normal stage (136 tests), and non-optimized state (101 tests). Table 4 provides the specific details and quantities of the tests conducted.

**Table 4:** Number of drilling tests on different types of rocks.

Rock group	Drilling state based on SE			No. of total tests
	Optimized stage	Normal stage	Non-optimized stage	
Granite	31	68	33	132
Marble	44	44	44	132
Travertine	24	24	24	72
Total tests	99	136	101	336

## Signal processing procedure and results

### Analysis of acoustic signals

**Fig. 7:**
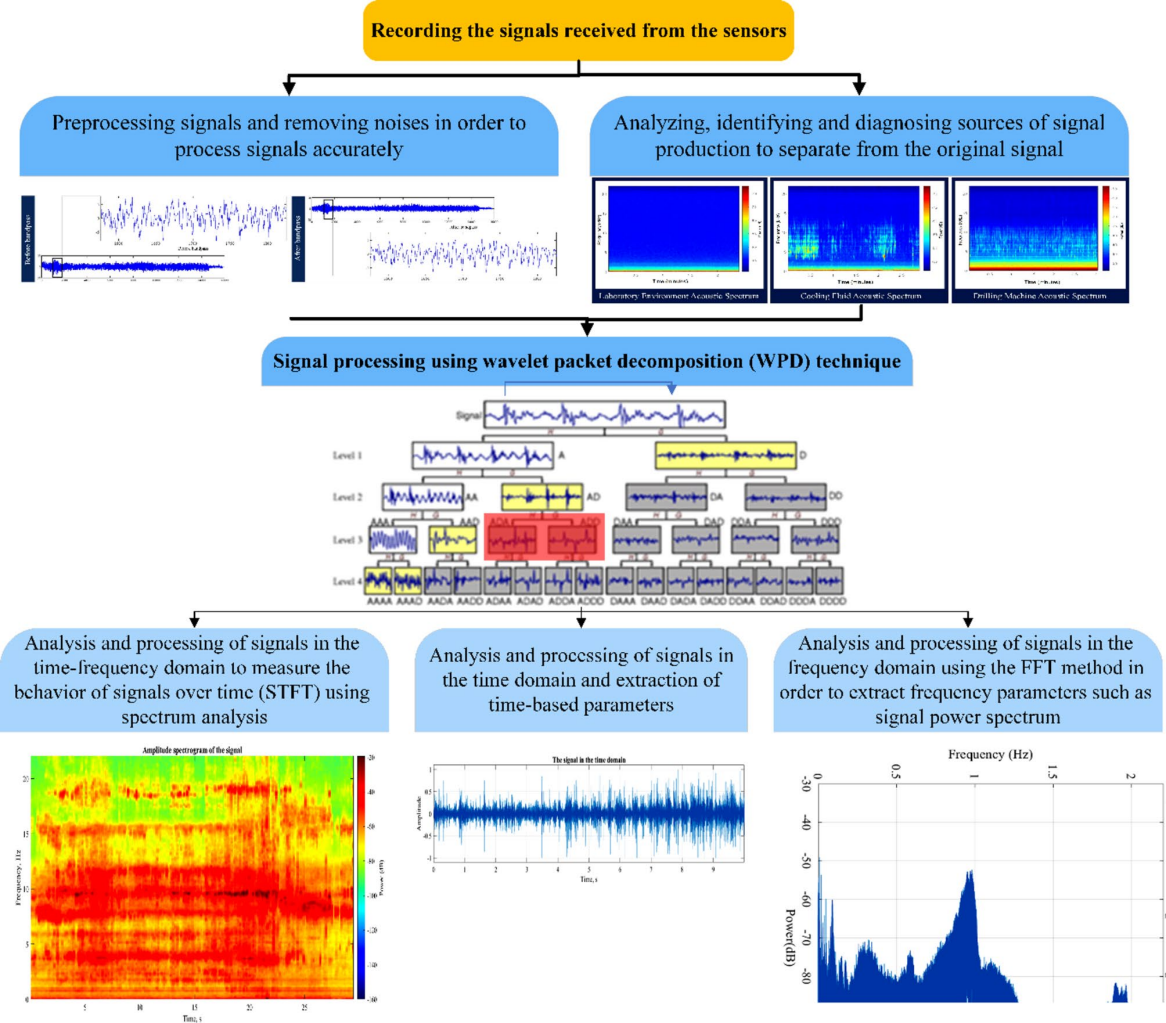
An overview of the used acoustic signal processing process.

Acoustic signals were recorded using the high-sensitivity microphone installed on the drilling machine. The signals were then processed using signal processing techniques to analyze time, frequency, and time–frequency domains.To fully comprehend and acknowledge the behavior of signals that are generated during rock drilling, various methods, and steps were employed to process, analyze, and extract the relevant features of these signals. Figure 7 gives an overview of the acoustic signal processing procedure.

Drilling vibroacoustic signals were processed using wavelet packet decomposition (WPD), time domain processing, frequency domain processing, and time–frequency spectrum analysis (as shown in Fig. 7). During the first stage of signal processing, the signal is evaluated and analyzed in the time domain to determine its amplitude at any given moment. This serves as a basic feature extraction method, allowing for statistical parameters to be extracted from the signal such as average amplitude, maximum amplitude value or peak, skewness, and other statistical parameters^55^. Some of the parameters that have been considered in the studies and evaluated in this paper are presented in Table 5.

**Table 5:** Commonly used parameters extracted from signals in the time domain.

Parameter	Equation	Definition
Crest factor	$Crest factor=max\left\{\left|{x}_{j}\right|\right\}/{T}_{rms}$	It is the ratio of the peak (peak) of a thirty wave to its effective value (RMS)^56^
Kurtosis factor	$Kurtosis factor=\left(\sum_{j=1}^{n}{\left({x}_{j}-{T}_{avg}\right)}^{4}\right)/\left(n.{T}_{sd}^{4}\right)-3$	Kurtosis means the sharpness of the peak, and the higher this value is, the sharper the peak is^56^
peak	$peak= max\left\{{x}_{j}\right\}$	It is the highest value of the signal during the entire signal
Skewness factor	$kewness facto\text{r}=\left(\sum_{j=1}^{n}{\left({x}_{j}-{T}_{avg}\right)}^{3}\right)/\left(n.{T}_{sd}^{3}\right)$	Skewness indicates the degree of asymmetry of the probability distribution of data around their mean. Skewness value can be negative or positive^56^
RMS (Root Mean square)	$RMS=\sqrt{\sum_{j=1}^{n}{x}_{j}^{2}}/n$	Expressing the root mean square of the signal values, which is also called the effective value of the signal^56^
Standard deviation	$Standard deviation=\frac{1}{n-1}\sqrt{\sum_{j=1}^{n}{\left({x}_{j}-{T}_{avg}\right)}^{2}}$	The standard deviation indicates the average distance of each value from the mean^56^
Count	n	The number of peaks of the signal wave that crosses certain thresholds

*In these equations: x_j_ is the signal value, T_rms_ is the effective value or RMS value of the signal, n is the number of data, T_avg_ is the average value and T_sd_ is the standard deviation of the signal values.

Frequency domain analysis is a method that overcomes the limitations of time domain analysis. By analyzing the frequency components of a signal, it is possible to extract important features while retaining a lot of information. To convert signals from their original time domain representation to their frequency domain representation, a Fast Fourier Transform (FFT) can be used. However, the main challenge of this method is to determine the spectral bands that are sensitive to drilling conditions, which is not always easy. Another limitation is the inability to understand why certain frequencies have an impact on tool wear. To determine which frequencies, dominate a signal's variance, one can calculate the power spectrum. The power spectrum describes how the intensity of a time-varying signal is distributed in the frequency domain.

In signal processing, when dealing with non-stationary signals, feature extraction is performed in the time–frequency domain. This approach addresses the limitations of single-domain analysis and is commonly used to generate features through the Short Time Fourier Transform (STFT). When processing signals that change rapidly over time, it is beneficial to consider the frequency content of short segments of the signal. The frequency content can be formulated as a two-dimensional function based on frequency and time position, resulting in a broader spectrum. A spectrogram is a crucial tool for analyzing time–frequency relationships in technical diagnostics^57^.

The Wavelet Transform (WT)is a technique used in signal analysis that divides a time domain signal into different frequency groups. Changes in time and frequency represent fluctuations in wave intensity. WPD is a more advanced version of wavelet decomposition (WP), which offers a wider range of possibilities for signal analysis and allows for the most appropriate analysis for a particular signal. This method transforms a signal from the time domain to the frequency domain level by level. By recursively applying filter-decimation operations, the time resolution is decreased, and the frequency resolution is increased^58^.

The WPD divides a signal into both low- and high-frequency sub bands, resulting in frequency bins of equal width. This differs from the WT, which involves convoluting a signal through high pass and lowpass filter pairs to split it into approximation and detail coefficients. To determine the next level of decomposition, the approximation coefficient is divided into second-level approximation coefficients and detail coefficients, and the process is repeated accordingly. However, WTs lack the resolution of the high-frequency signal components, as they decompose signals only on the lower half of the bandwidth. Both the details and approximations can be further split into wavelet packet analysis. When the WD is generalized to the WPD, not only can the lowpass filter output be iterated through further filtering, but the high pass filter can also be iterated. If all lowpass and high pass filters are iterated, the complete tree basis results^59^.

#### Identification of acoustic signal frequencies and amplitudes related to rock drilling.

**Fig. 8:**
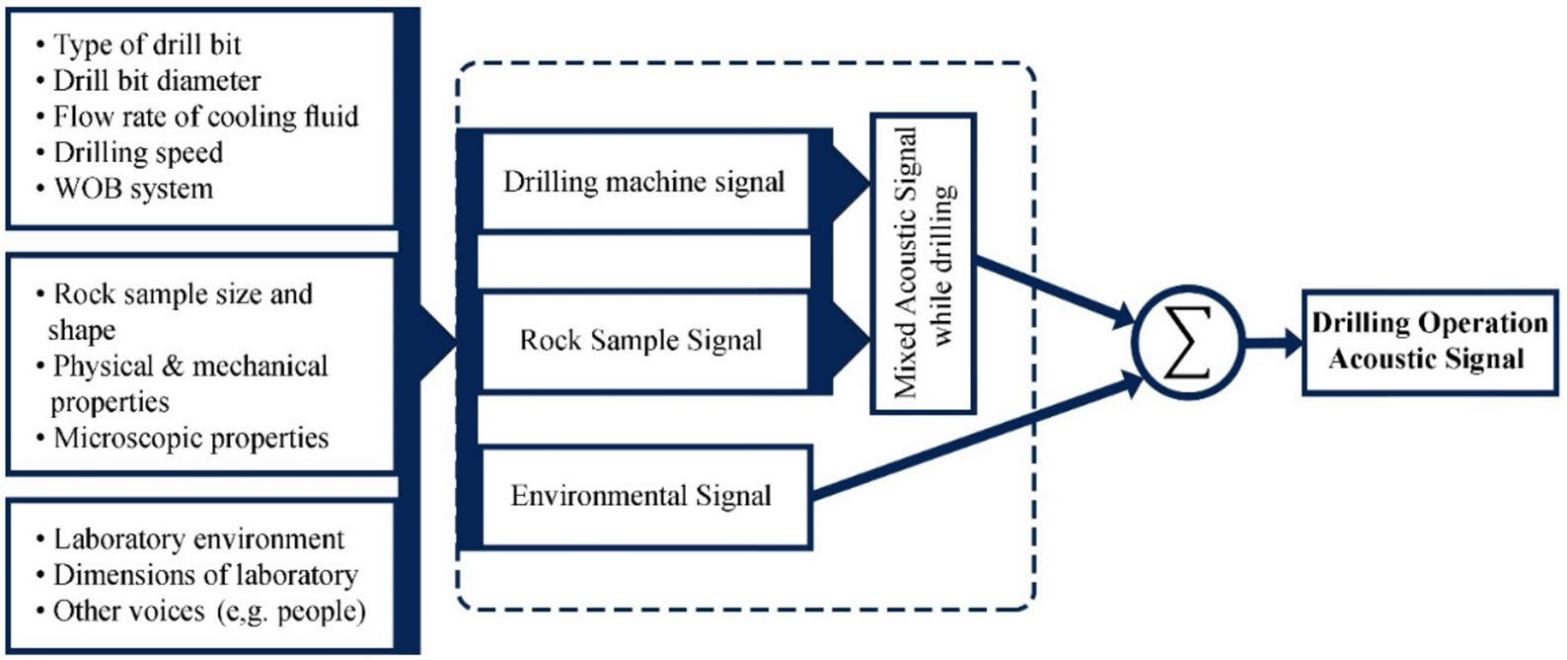
Classification of acoustic signal sources and factors affecting drilling operations in a laboratory^50^.

During a drilling operation, the acoustic signal detected is a combination of signals from various sources. As depicted in Fig. 8, these sources can be categorized into three broad classes. The figure also highlights the factors that influence the properties of acoustic waves.

**Fig. 9:**
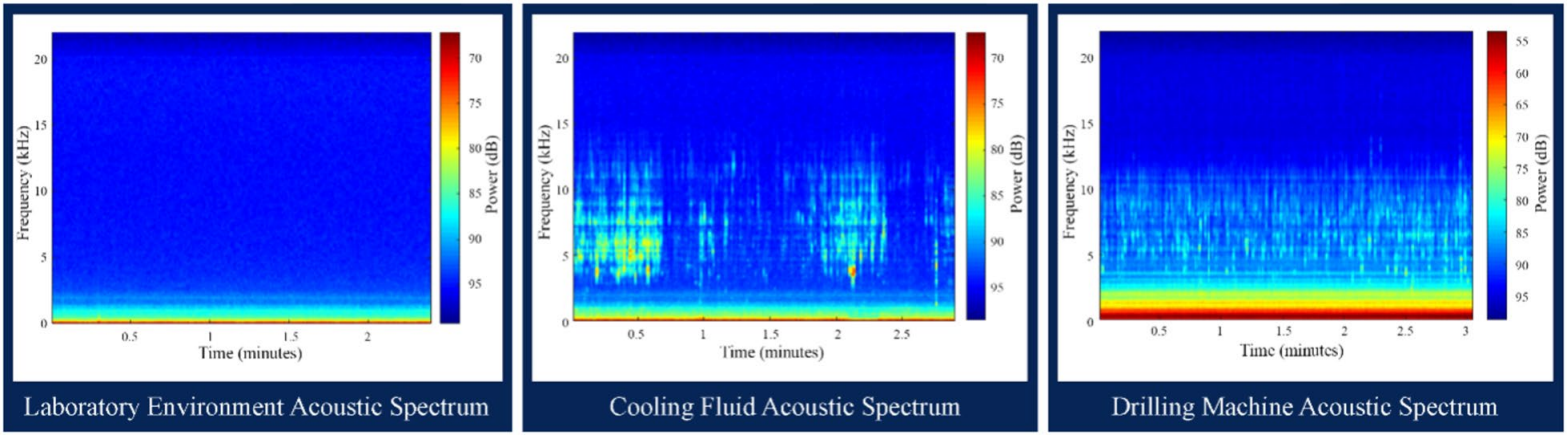
Frequency-time spectra of the different laboratory conditions before the drilling tests.

To determine the frequency range that relates to the interaction between a drill bit and rock, it's essential to record and process the acoustic signals in various laboratory conditions. These conditions include the laboratory environment noise, drilling machine sound, and drilling machine sound in the presence of drilling fluid (Fig. 9). The collected acoustic data undergoes analysis through STFT, which allows obtaining the intensity at each frequency and the duration of its presence in the signal spectrum^60,61^.

The frequency ranges of the laboratory environment, drilling fluid, and drilling machine are extracted using the spectra displayed in Fig. 9. Table 6 provides the frequency range and maximum amplitude of these sources. To determine the signal frequency range, the cut-off value was set at 50% of the maximum signal amplitude. Note that this level is only for representation purposes and has no effect on further analysis. Table 4 shows that the maximum frequency from sources other than the interaction between the drill bit and rock samples is 1500 Hz.

**Table 6:** Maximum amplitude and frequency range of the different laboratory conditions before the drilling trials.

Signal recording mode	Maximum signal amplitude (dB)	Signal frequency range (Hz)
Laboratory environment	− 73	0–200
Cooling fluid	− 73	0–1200
Drilling machine	− 53	0–1500

#### Analysis of acoustic signal spectra from drilling in the rock samples

**Fig. 10:**
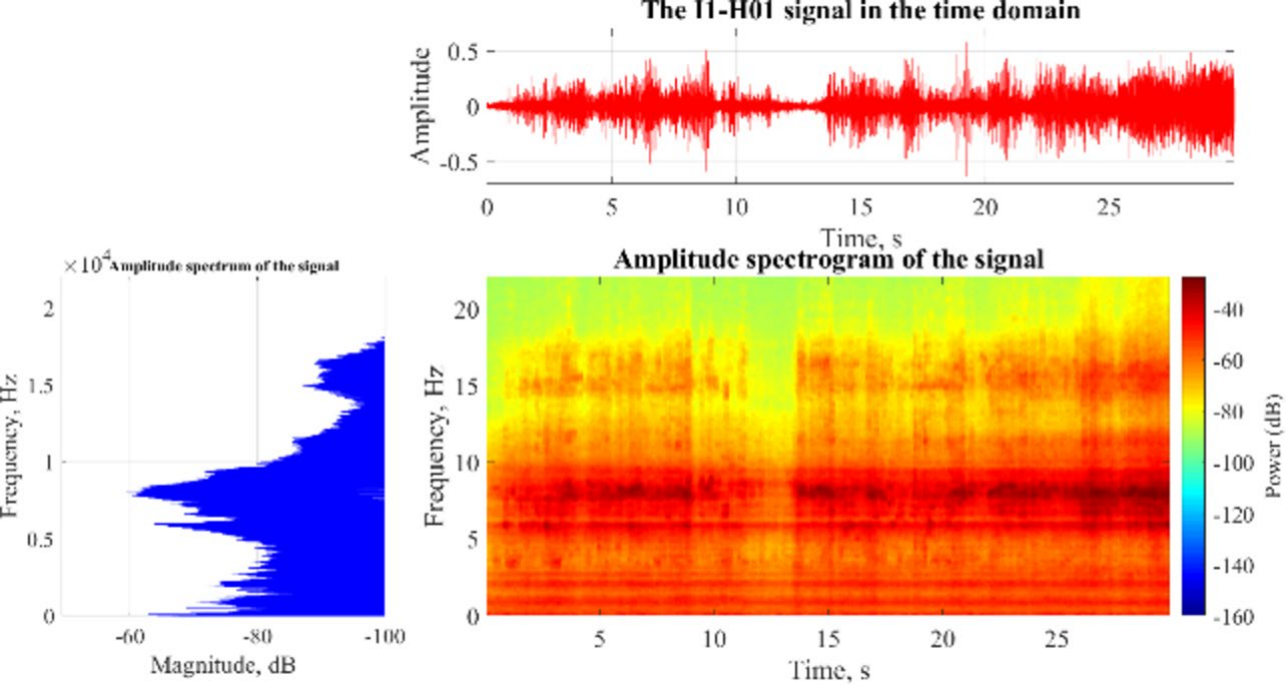
An example of the recorded acoustic signal for sample #1 in the time domain (top), in the frequency domain (left), and in the two-dimensional time–frequency domain (right).

The acoustic signals obtained during the drilling of rocks are analyzed in three different ways. Firstly, the signals are presented in a single domain to obtain parameters related to the time domain. This information is then used to conduct spectral analysis of the signals by FFT, which extracts the dominant frequencies and their power density. Secondly, a two-dimensional time–frequency spectral analysis by STFT is performed to determine the frequency range related to the rock characteristics and to compare the different acoustic spectra during drilling. Lastly, the WPD technique is used to separate the considered frequency bands from other parts of the signal. To illustrate, Fig. 10 shows the acoustic signal in the time domain for the first sample, along with its frequency and time–frequency spectra.

After evaluating and analyzing the acoustic signals obtained from drilling rock samples under various conditions, it was observed that there is a high-energy frequency band present in all signals. This frequency band exists in all rocks and drilling energy consumption conditions and is independently related to the rock properties and drilling conditions. The frequency range of 6500–10,000 Hz is particularly important in this regard. It is not contaminated by other acoustic sources, as the energy from other sources is orders of magnitude less than the acoustic signal generated by the interaction of the drill bit and rock samples. This frequency range is therefore used to extract the dominant frequencies in subsequent examinations.

**Fig. 11:**
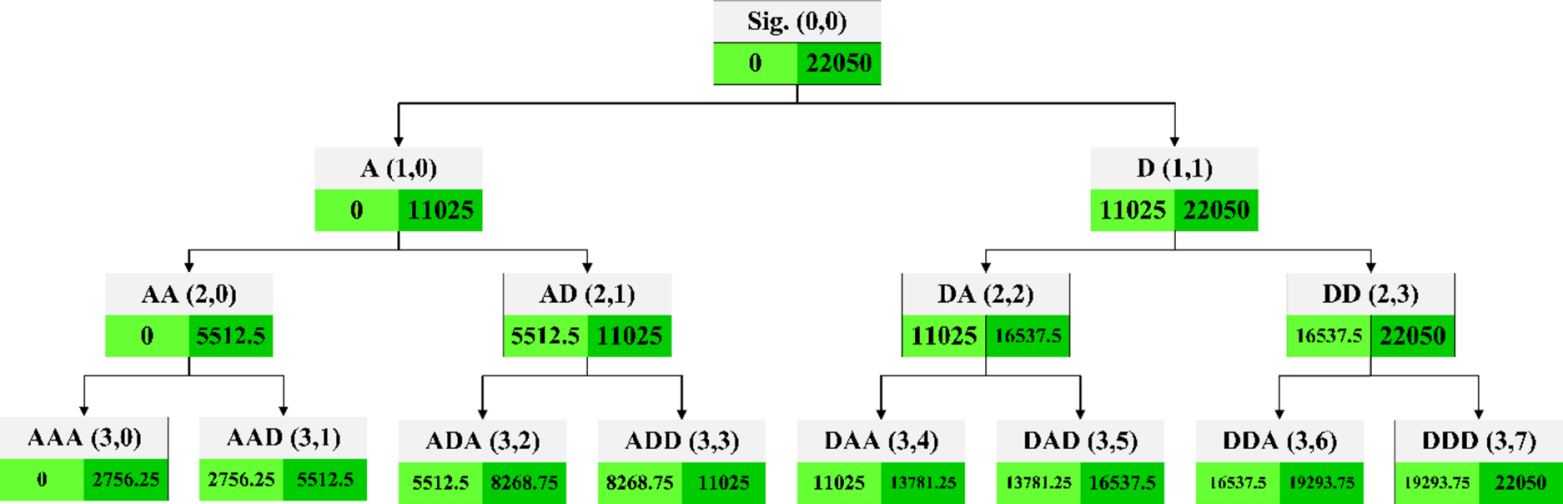
Results of analysis of WPD and wavelet packets relate to each sound source.

An analysis of signal spectrums was conducted under various rock drilling conditions to determine the frequency range. It was observed that the (3,2) and (3,3) packets are affected by drilling conditions, while machine background noise corresponds to the (3,0) packet. To enhance drilling energy and mitigate machine noise, sound signal features were extracted from the (3,2) and (3,3) packets instead of the primary time-domain signal (Fig. 11). By extracting time domain features (refer to Table 5) from the wavelet packet decomposition tree's (3,2) and (3,3) nodes, signatures for the analyzed acoustic signals were identified.

**Fig. 12:**
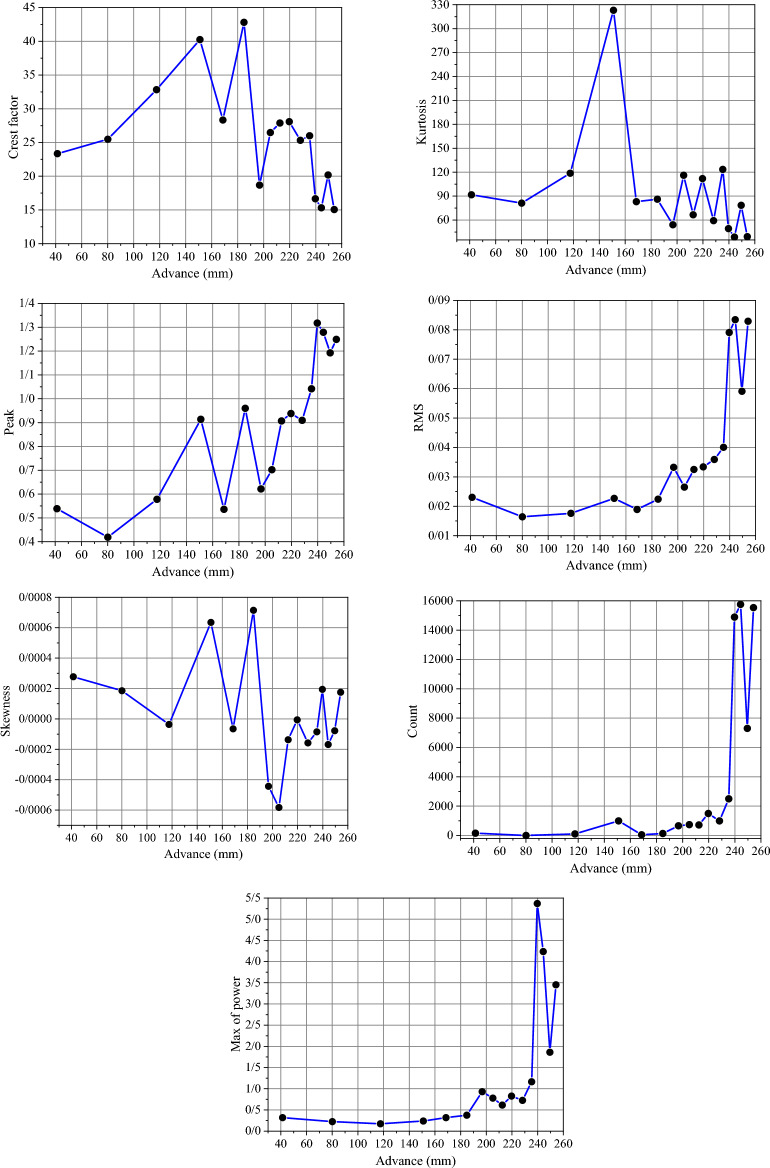
Changes in the parameters of the acoustic signals of the drilling of rock sample I1 during the lifetime of the drill bit.

The analysis of acoustic signals in the time–frequency domain helped identify the segments of the signal that were related to the drilling conditions and the interaction between the drill bit and rock. The WPD method was then employed to focus on the key parts of the signal that indicated the drilling conditions. By separating the signal parts related to the interaction of the drill bit and rock, the acoustic signals of each rock were analyzed from the start to the end of the drill bit's life. To achieve this, parameters based on time and frequency, explained in the previous sections, were extracted from each signal, and compared with one another in each rock sample under different drilling conditions. Figure 12 shows the process of changes in the parameters of the drilling acoustic signals of rock sample I1 in the domains of time and frequency.

**Fig. 13:**
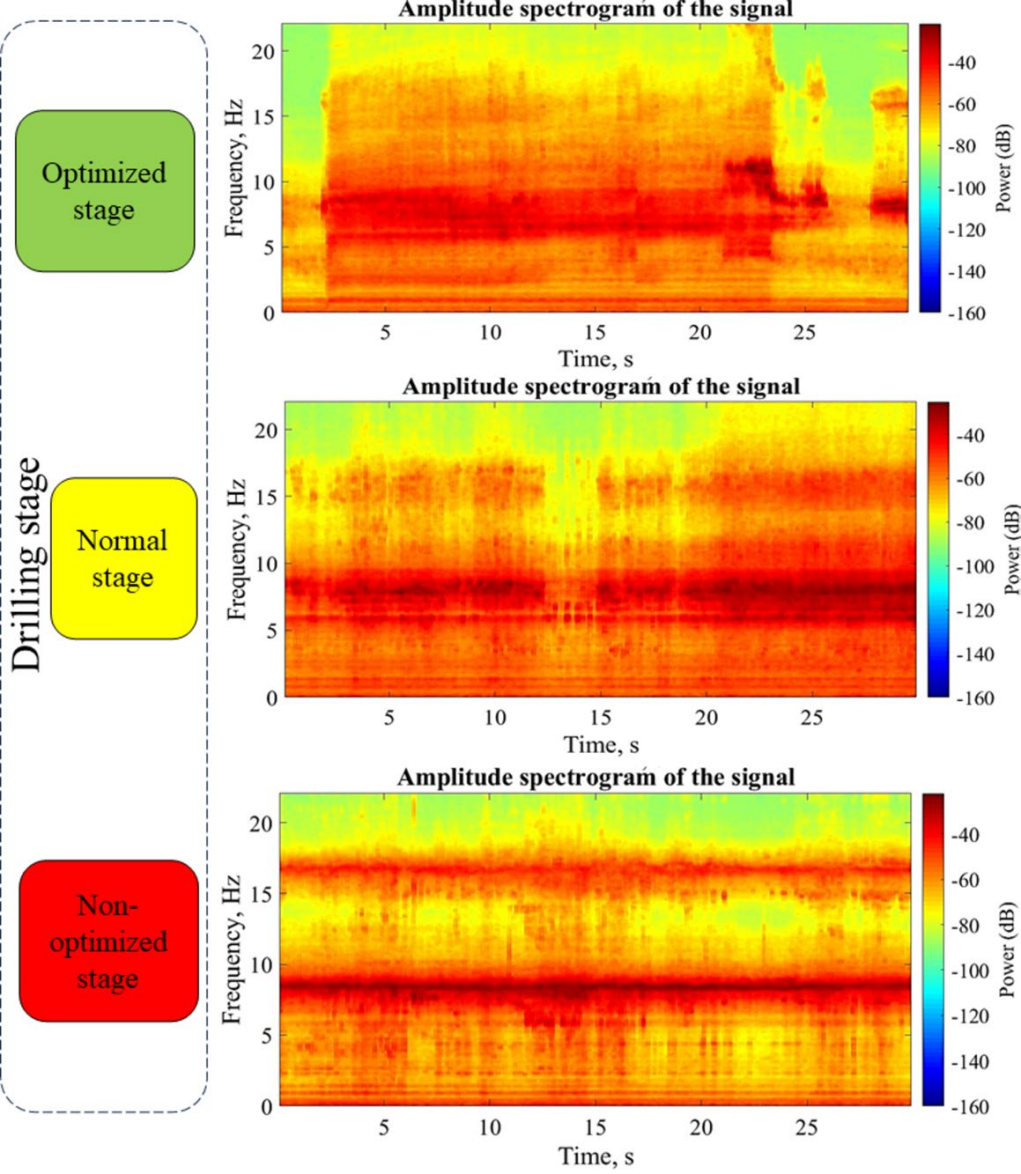
Changes in the energy intensity of the frequency band related to rock drilling of acoustic signals in different stages of drilling.

The graphs in Fig. 12 show the high sensitivity of acoustic signals to changes in drilling conditions. So that it is possible to measure changes in drilling conditions with high accuracy and speed with acoustic signals. The changes in the acoustic parameters shown in the graphs of Fig. 12 are completely consistent with the change in the SE behavior during the drilling tests. So, during non-optimal drilling conditions, the acoustic signals exhibit higher intensity and energy. Figure 13 provides an example of changes in sound energy during drilling tests conducted under optimal, normal, and non-optimal conditions, in the frequency range related to rock drilling.

#### Analysis of vibration data (3-axis accelerometer) from the drilling tests

##### Nature of vibration data

**Fig. 14:**
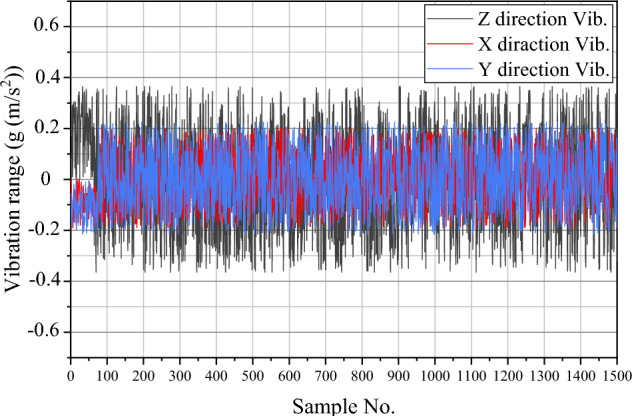
The time spectrum for the vibration signals in the direction of the x, y, and z axes for sample #1.

During the drilling operation, vibration waves are generated and transmitted to the drilling machine. These waves are recorded by a 3-axis accelerometer which outputs the vibration acceleration of the machine in m/s^2^^62^. To minimize errors in sensor recordings, the accelerometer is positioned exactly on the axis of the drill bit, reducing the torque created at the top of the machine. An example of the time variations of the vibration signal in the x, y (lateral vibrations), and z (axial vibration) axes can be seen in Fig. 14 (the horizontal axis represents the recorded samples).

##### Signal pre-processing

**Fig. 15:**
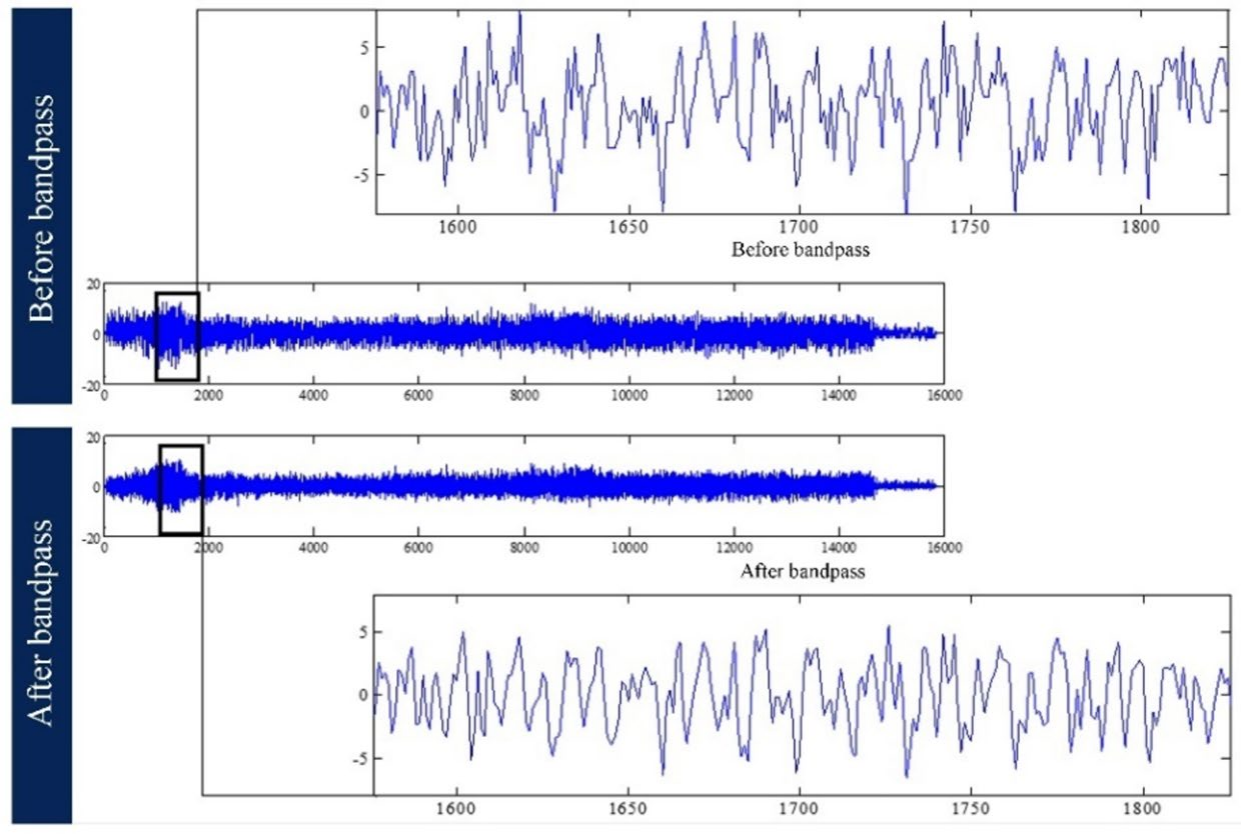
Comparison of a vibration signal before and after applying a bandpass filter between 2 and 20 Hz.

After recording the vibration signals, the first step is to pre-process them to prepare the vibration signal or spectrum for the main processing and frequency analysis. To do this, a bandpass filter in the frequency range of 2–20 Hz is applied. This range is chosen because the frequencies below this range are only related to the vibration of the drilling machine, and those above it are noisy. The purpose of this step is to eliminate the noise from the vibration signals, which are frequencies that are not directly related to the drilling of rocks. A recorded vibration signal is compared before and after this filtering in Fig. 15.

##### Processing of vibration signals

**Fig. 16:**
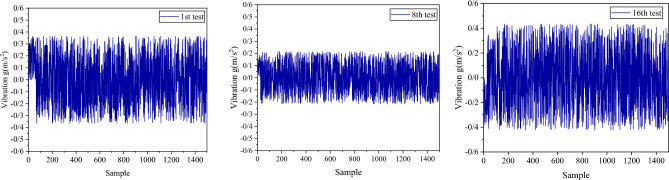
Vibration signals in Z direction (axial vibration) in rock sample I1 in three stages of drilling.

Based on previous studies, two parameters—the maximum amplitude value (peak) and the root mean square value (RMS or effective acceleration)—have been extracted from each vibration signal to determine the energy condition of the system^63^. These two parameters are used to evaluate the relationship between the vibration signals generated during drilling and the drilling SE. After investigating the vibration signals in various tests, it was observed that the vibration values are relatively high when using a new drill bit. However, as the drill bit ages, the vibration values decrease and follow a uniform trend. On the other hand, during the non-optimal drilling stage (characterized by high SE values), a significant increase in vibration values is observed. This issue can be easily observed in the time spectra of the signals. To illustrate this issue further, Fig. 16 displays the vibration in the Z axis for the first, eighth, and sixteenth tests in sample No. I1.

## Results and discussion

After determining the SE of each drilling test, the properties of the recorded vibroacoustic signals were extracted, and signal processing was conducted to investigate the correlation between drilling SE and observed vibroacoustic signal properties. There are several ways to process acoustic signals, including Fourier transform, Wavelet transform, Hilbert-Huang transform, and others. Each of these methods has a unique mathematical approach and provides different results. This study used WPD, FFT, and STFT methods, which are among the most widely used, most accurate, and simple signal processing methods.

Using the STFT method, frequency intervals in acoustic signals from different sources like drilling engines or rock drilling can be identified after analyzing the signals in the time domain and determining the frequency range of the rocks using the FFT method, the power of dominant frequencies with the highest energy intensity calculated as the signal's characteristics. These characteristics were then investigated for their relationship with the drilling SE.

**Fig. 17:**
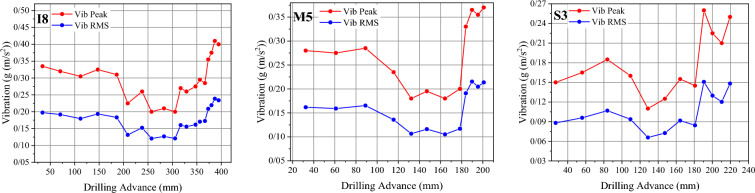
Maximum and RMS changes of vibration signals during drilling tests of three rock samples.

The analysis of acoustic signals during rock drilling has shown that using certain acoustic features can help identify areas of non-optimized drilling. This study used vibration signals to distinguish between optimized, normal, and non-optimized conditions. When the drill bit was fresh (in the optimized region), high peak and RMS values were observed at the beginning of the drilling operation. During the normal phase, vibration features decreased and reached a minimum value. However, during the non-optimized phase, drilling SE increased, promoting vibration characteristics. As a result, peak and RMS values reached their maximum in the non-optimized phase (Fig. 17).

Using the technique of processing vibro-acoustic signals during drilling operations is a smart and innovative method in monitoring drilling operations. In this paper, the possibility of using this method was investigated. The investigations have shown that it is possible to monitor and calculate the SE of drilling tests with high accuracy by processing the signals using the vibroacoustic signal characteristics generated during the drilling operation.

This method removes the limitations of previous methods and can be employed with simple equipment to collect acoustic and vibration signals. Compared to existing methods, this method offers an accurate and real-time monitoring solution that can be easily integrated with existing drilling equipment. The collected signals can then be coded and statistically processed with high accuracy and speed and at the lowest cost.

## Conclusions

This study explores the relationship between SE of drilling and the characteristics of vibroacoustic signals produced during drilling operations in mines. The energy consumption of drilling operations is a crucial factor in drilling monitoring and optimizing, and the use of vibroacoustic signals is gaining significance in the field of engineering. To conduct this study, a laboratory-scale rotary drilling machine was equipped with signal recording equipment. Drilling tests were conducted on 30 various rock samples and diverse lithological origins, including igneous, metamorphic, and sedimentary rocks. Throughout each drilling test, vibroacoustic signals such as drilling sound and machine vibration were recorded. Following the drilling tests, the signals generated during the operation were processed in the time and frequency domain using the FFT method. Additionally, a 2D analysis of time–frequency signals was conducted using the STFT and WPD methods. Parameters were extracted for each area to facilitate the analysis, and their correlation with the SE was evaluated. The results obtained from signal processing and evaluations are as follows:

- The frequency band of each signal source was determined by processing the acoustic signals and extracting their frequency content to remove the noises and other signals sources. The frequency range related to the drilling environment, drilling fluid, and the drilling machine was obtained from 0 to 1500 Hz. The process of interaction of drill bit and rock affects the frequency band of 6500 to 10,000 Hz. Therefore, the processes performed and the extraction of signal characteristics from this band provide helpful information for predicting the SE and for condition monitoring of drilling operations. For this purpose, signal analysis and feature extraction from the appropriate node performed using the WPD technique. This helps the effective and correct processing of the signals for the real-time prediction of the SE.
- By starting the drilling operation on each rock sample with the same operating parameters, from the beginning (optimal mode of drilling with a new bit) to the end, the life of the bit and its replacement time (non-optimal drilling mode) continuously increased energy consumption. The exact process observed in the behavior of acoustic and vibration signals. With the progress of drilling and the wear of the drill bit, the sound intensity also showed an increasing trend. As the drill bit nears the replacement time, as the energy consumption has suddenly increased and drilling is done at a very low penetration rate, the acoustic signals also increase in this area.
- The vibration signals of the rock drilling operation were examined alongside the acoustic signals. Two parameters, signal peak and root mean square (RMS), were obtained from each vibration signal and analyzed. The changes in these two parameters were also like the changes in drilling SE, with the difference that after the drilling was done from the beginning and the bit passed through the sharp state, the vibration signal decreased and increased upon reaching the non-optimal drilling stage. This issue shows that drilling vibration signals are a valuable tool in drilling condition monitoring and energy consumption state detection.

Using this method allows for real-time measurement of drilling behavior, enabling the optimization of drilling operations by identifying energy consumption and ROP. This method also helps detect non-optimal drilling times and promptly recognizes drill bit failure, allowing for the intelligent replacement of the bit and optimization of drilling operations. Real-time measurement of drilling behavior using this method can greatly enhance drilling performance and aid in automation. Additionally, drilling monitoring is useful for the machining industry and the future development of Industry 4.0.

## Data Availability

All data generated or analyzed during this study are included in this published article (and its Supplementary Information files).
